# Blockchain for Digital Health Governance: Evidence Gap Map and Scoping Review

**DOI:** 10.2196/96098

**Published:** 2026-10-07

**Authors:** Sarina Yaghobian, Nina Sulkowski, Gary Galambos, Linda Scarazzini, Nicolas Maloumian, Stephane Verhaeghe

**Affiliations:** 1 Télémédecine 360, TLM360 SAS,, 55 Avenue Marceau, Paris, Ile-de-France, 75116, France, 33 643610937; 2 Independent researcher, Sydney, NSW, Australia; 3 Department of Psychiatry, The University of Notre Dame Australia, Sydney, NSW, Australia; 4 Lewis Katz School of Medicine, Temple University, Philadelphia, PA, United States; 5 School of Medicine, Adelaide University, Adelaide, South Australia, Australia

**Keywords:** blockchain, distributed ledger technology, digital health, data governance, scoping review, evidence gap map, interoperability, health information exchange

## Abstract

**Background:**

Digital health increasingly depends on data exchange across institutions, technologies, and jurisdictions, creating persistent challenges for the governance of access, consent, interoperability, provenance, and accountability. Blockchain and distributed ledger technology (DLT) systems have been proposed as mechanisms for coordinating and verifying governance processes across distributed actors. However, existing research has examined individual applications or technical domains, leaving unclear how blockchain/DLT systems function as governance infrastructure across health care, and whether these systems have progressed toward real-world implementation.

**Objective:**

This evidence gap map and scoping review aim to characterize how blockchain/DLT systems are applied to health data governance, identify the health care application domains, and governance functions addressed by these systems, and assess their evidence maturity.

**Methods:**

We searched PubMed/MEDLINE, Embase, Scopus, and Dimensions (Digital Science), and conducted backward and forward citation searching to identify peer-reviewed studies reporting blockchain/DLT systems in health care with an implemented artifact, technical evaluation, simulation, benchmark, pilot/usability assessment, or operational deployment, published between January 1, 2010, and February 28, 2026. Conceptual or architecture-only papers were excluded. Studies were charted by application domain, governance function, and evidence maturity (proof-of-concept/prototype, simulated/benchmarked evaluation, pilot/usability-tested implementation, or operational/real-world deployment). A focused narrative synthesis was conducted for studies reporting pilot/usability-tested implementation or operational/real-world deployment.

**Results:**

Of 892 included studies, the evidence base was dominated by proof-of-concept/prototype (n=418) and simulated/benchmarked evaluation (n=455) work; only 18 reported pilot/usability-tested implementation, and 1 reported operational/real-world deployment. Studies were concentrated in electronic health record management/health information exchange (n=372) and telemedicine/distributed care/Internet of Things (IoT)–enabled remote monitoring (n=250), followed by clinical decision support/smart health care (n=82), public health surveillance/certification (n=72), clinical trials/research governance (n=67), and health data marketplace/monetization (n=49). Across 3273 nonmutually exclusive governance-function codes, the most frequent functions were privacy/security, interoperability/data sharing, access control, data/model integrity, and identity/authentication. Publication activity increased over time, with the highest annual volumes in 2022 and 2025. This growth was not accompanied by a shift toward higher-maturity evidence. Privacy/security and interoperability/data sharing remained prominent across publication years, while other governance functions varied over time. Higher-maturity evidence was unevenly distributed across application domains, particularly clinical trials/research governance and electronic health record management/health information exchange. Within the 19 higher-maturity studies, evidence remained limited by small-scale evaluations, short follow-up, and a lack of sustained routine use beyond the evaluation period.

**Conclusions:**

Across digital health, blockchain/DLT systems were positioned as governance infrastructure for verifiable access, consent, provenance, identity, and audit trails, rather than as repositories for health data. Despite a growing literature, real-world implementation evidence remained limited. Whether blockchain/DLT systems improve governance outcomes over conventional architectures remains largely untested. Future research should prioritize comparative, implementation-focused evaluation of whether these systems can be integrated, sustained, and shown to provide governance benefits in real-world settings.

## Introduction

The rapid digitalization of health care has transformed how health information is generated, exchanged, and reused across clinical care, research, and public health. Electronic health records (EHRs), remote monitoring technologies, connected devices, and AI-enabled analytics have expanded opportunities for data sharing and coordination. However, their use has brought challenges related to interoperability, trust, and accountability into sharper focus [1]. Health information remains fragmented across institutions and jurisdictions, complicating data exchange and dispersing responsibility for access, consent, and oversight. Regulatory initiatives such as the European Health Data Space (EHDS) reflect growing recognition that secure and accountable secondary use of health data depends on effective governance [2,3].

These challenges also extend beyond technical considerations. Digital health systems depend on patients’ ability and willingness to engage with their care and their trust in how their data are used. This is particularly relevant to chronic disease management, a major application of remote monitoring and other digital health technologies [4]. In a recent Organisation for Economic Co-operation and Development (OECD) survey examining people-centered and coordinated care across 19 countries, 58.9% of primary care patients living with chronic conditions reported feeling confident managing their own health [5]. Trust in digital health, shaped by concerns about privacy, data use, accuracy, and accountability, remains a key determinant of adoption [6]. Together, these considerations reinforce the need for clear rules governing who may access health data, for what purposes, under what conditions, and through which mechanisms of oversight.

Governance, as used here, refers to the rules, processes, and accountability mechanisms that shape how health data are accessed, shared, controlled, and monitored across actors and organizations [7-9]. Proprietary platforms and siloed infrastructures continue to impede data sharing [8,9], while the increasing use of health data for secondary purposes raises concerns about transparency, consent, oversight, and patient control. Effective governance must therefore balance data access for care, research, and innovation with legal, ethical, and privacy obligations.

Distributed ledger technology (DLT) systems, including blockchain, have been proposed as a means of supporting some of these governance functions [10]. Blockchain systems create tamper-evident, time-stamped records and can support programmable rules for data access and exchange [11]. These properties have prompted applications in consent management, access control, identity verification, provenance tracking, auditability, and cross-organizational coordination [12-14]. Although pilot implementations have demonstrated technical feasibility in areas such as health information exchange, clinical trials, and patient-controlled data sharing, evidence of sustained real-world implementation remains limited [15].

A substantial body of literature has examined blockchain applications in health care [16,17] and within specific areas, including EHR interoperability [18], patient care [19], design choices and implementation trade-offs [20], genomics [21], consent management [22], and COVID-19-related applications [23]. Recent reviews have assessed health care blockchain implementation and performance more generally [24]. However, existing reviews remain largely organized around specific applications, clinical domains, or technical architectures. Governance functions are generally discussed within individual use cases rather than systematically compared across digital health contexts, and their relationship with evidence maturity remains unclear.

We therefore conducted an evidence gap map and scoping review of blockchain/DLT applications in digital health governance. The objectives of this study were to identify recurring governance functions, map evidence maturity across health care domains, and highlight priorities for future research, implementation, and policy.

## Methods

### Protocol and Registration

This scoping review was reported in accordance with the PRISMA-ScR (Preferred Reporting Items for Systematic Reviews and Meta-Analyses extension for Scoping Reviews; Checklist 1) [25]. The search strategy was reported in accordance with the PRISMA-S (Preferred Reporting Items for Systematic Reviews and Meta-Analyses literature search extension) to improve transparency and reproducibility [26]. A scoping review design was selected given that the literature on blockchain/DLT systems in digital health is heterogeneous. A protocol was not registered.

We aimed to map and synthesize peer-reviewed literature on blockchain/DLT systems in digital health governance. Specifically, we examined recurring governance functions, implementation patterns, evidence maturity, and limitations across health care application domains. Governance-layer infrastructure was defined as systems that record, verify, or coordinate permissions, access events, consent decisions, provenance, and audit trails across distributed actors, rather than storing clinical data. In this study, “on-chain” refers to information recorded directly on a blockchain/DLT, whereas “off-chain” refers to data stored outside the ledger, such as in clinical databases, institutional repositories, cloud storage, or distributed file systems. In these architectures, the ledger typically records hashes, permissions, metadata, transactions, or consent events.

### Eligibility Criteria

Eligibility criteria were structured using the population/concept/context approach recommended for scoping reviews [25]. Studies were included if they (1) examined blockchain/DLT systems, smart contracts, or decentralized ledger infrastructure; (2) were situated within health care, medicine, public health, digital health, telemedicine, clinical research, AI in health, EHR, or health data exchange; (3) addressed at least one governance-related function, including data sharing, consent management, access control, auditability, accountability, identity management, provenance, interoperability, regulatory compliance, data stewardship, or cross-organizational coordination; (4) reported an implemented or evaluated system, including a prototype, simulation, benchmark, pilot, usability study, evaluation study, or operational implementation; and (5) were peer-reviewed publications in English published within the specified date range. Reviews were used to inform the background, search strategy, and backward and forward citation searching but were not included as primary evidence records. Gray literature was excluded to support reproducibility and consistency in eligibility assessment, given the substantial variation in reporting detail, methodological transparency, and implementation verification across nonpeer-reviewed sources.

Studies were excluded if they (1) did not examine blockchain, DLT, smart contracts, or decentralized ledger mechanisms; (2) were not situated within a relevant health care or digital health context; (3) focused exclusively on cryptocurrency, finance, nonhealth care supply chains, logistics, education, or other nonhealth care applications; (4) focused solely on cryptographic design, consensus algorithms, performance optimization, or technical engineering without relevance to governance; (5) did not address any governance-related function; (6) were conceptual, theoretical, or architecture-only papers that did not report an implemented artifact, simulation, benchmark, pilot, usability assessment, evaluation, or operational deployment; (7) were editorials, commentaries, opinion pieces, news items, dissertations, theses, preprints, white papers, book chapters, conference abstracts without sufficient detail, or other nonpeer-reviewed sources; (8) were retracted, withdrawn, or otherwise invalid publications; or (9) were not published in English or fell outside the inclusion date range.

### Information Sources

Searches were conducted in PubMed/MEDLINE, Embase via Ovid, Scopus, and Dimensions (Digital Science). Searches were limited to English-language publications from January 1, 2010, to February 28, 2026. Peer-review status was assessed during eligibility screening. The final database searches were conducted on May 25, 2026.

Supplementary searching was conducted through backward and forward citation searching. Backward citation searching involved screening the reference lists of included studies and relevant review articles. Forward citation searching was conducted using Dimensions to identify citing articles. Records identified through citation searching were deduplicated and screened using the same eligibility criteria as database-derived records.

### Search

The search strategy was developed using controlled vocabulary terms and free-text synonyms for three core concepts: (1) blockchain/DLT systems; (2) health care and digital health contexts; and (3) governance-related functions. Search terms were informed by seed articles, prior reviews, and terminology identified in titles, abstracts, index terms, and author keywords.

Across databases, the search used synonyms and related terms for blockchain technologies, including “blockchain,” “distributed ledger,” “distributed ledger technology,” “smart contract,” “decentralized identity,” “self-sovereign identity,” “tokenization,” and “Web3”; health care terms, including “digital health,” “eHealth,” “mHealth,” “telemedicine,” “telehealth,” “electronic health records,” “clinical trial,” “biomedical research,” “artificial intelligence,” “machine learning,” and “health data”; and governance-related terms, including “data governance,” “consent,” “dynamic consent,” “access control,” “audit trail,” “auditability,” “provenance,” “traceability,” “interoperability,” “privacy,” “security,” “accountability,” “regulation,” “regulatory compliance,” “data stewardship,” “data sharing,” “data exchange,” and “health information exchange.”

PubMed/MEDLINE and Embase searches via Ovid combined controlled vocabulary terms, including MeSH and Emtree terms, along with free-text terms. Scopus searches were conducted in the title, abstract, and keyword fields, and Dimensions searches were conducted in the title and abstract fields. Fully reproducible database-specific search strategies, including field tags, controlled vocabulary terms, date and language limits, search dates, and records retrieved, are provided in Multimedia Appendix 1. The overall database search structure is summarized in Multimedia Appendix 2.

### Selection of Sources of Evidence

All records identified through database searching and supplementary citation searching were imported into Rayyan (Qatar Computing Research Institute) for deduplication and screening. Duplicate records were removed before title and abstract screening. Two reviewers independently screened titles and abstracts against the eligibility criteria. Records that met the inclusion criteria, or for which eligibility could not be determined from the title and abstract alone, were retained for full-text review.

Full-text articles were independently assessed by the same reviewers. During full-text screening, records were also checked for retraction, withdrawal, or an invalid publication status, and any such records were excluded. Records identified through backward and forward citation searching were screened using the same eligibility criteria as database-derived records. Disagreements at both screening stages were resolved through discussion and consensus. The study selection process, including the numbers of records identified, screened, assessed for eligibility, excluded, and included, is reported in the PRISMA (Preferred Reporting Items for Systematic Reviews and Meta-Analyses) flow diagram.

### Data Charting Process

A standardized data charting form was developed and piloted on a subset of included studies. The form was refined during piloting to ensure that it captured both descriptive study characteristics and governance-related information relevant to the review objectives. Data charting was performed by one reviewer and checked by a second reviewer for completeness and consistency. Discrepancies were resolved through discussion and consensus.

### Data Items

Extracted data included bibliographic information, year of publication, publication type, study design, health care or digital health context, application domain, governance challenge addressed, blockchain/DLT mechanism described, on-chain or off-chain architecture, where reported, implementation status and follow-up, evaluation methods, reported outcomes, limitations, and relevance to cross-domain governance functions. Evidence maturity was categorized as proof-of-concept/prototype, simulated/benchmarked evaluation, pilot/usability-tested implementation, or operational/real-world deployment.

### Critical Appraisal of Individual Sources of Evidence

A formal risk-of-bias assessment was not conducted because the included literature was methodologically heterogeneous and consisted largely of system design studies, prototypes, simulated/benchmarked evaluations, and early-stage pilot implementations for which standard risk-of-bias tools are not typically applicable. Consistent with the purpose of a scoping review, studies were not excluded on the basis of methodological quality.

### Synthesis of Results

Data were synthesized using a thematic approach. First, studies were mapped by health care application domain, governance function, and evidence maturity to develop the evidence gap map. This mapping was used to identify areas with concentrated evidence, areas dominated by proof-of-concept/prototype studies or simulations/benchmarked evaluations, and areas where pilot/usability-tested implementation and operational/real-world deployment evidence remains limited.

Because the mapped evidence base was large and heterogeneous, the focused narrative synthesis was restricted to studies reporting pilot/usability-tested implementation or operational/real-world deployment. Proof-of-concept/prototype studies and simulated/benchmarked evaluations were retained in the evidence gap map but were not included in the focused narrative synthesis.

## Results

### Study Selection

Database searches identified 12,016 records: 1727 from PubMed/MEDLINE, 2827 from Embase via Ovid, 3777 from Scopus, and 3685 from Dimensions (Figure 1). After the removal of 4916 duplicate records, 7100 records were screened by title and abstract. Of these, 5552 were excluded, leaving 1548 full-text articles for eligibility assessment. All 1548 full-text articles were retrieved and assessed.

Following full-text assessment of the database-derived records, 888 studies met the inclusion criteria. Backward and forward citation searching identified 145 additional records. After deduplication and screening, 6 full-text articles were assessed, of which 4 met the inclusion criteria and 2 were excluded. Overall, 892 studies were included in the evidence gap map. Of these, 19 studies reported pilot/usability-tested implementation or operational/real-world deployment and were included in the focused narrative synthesis. The complete list of included studies and their classifications by health care application domain, governance function, and evidence-maturity category is provided in Multimedia Appendix 3.

**Figure 1.**
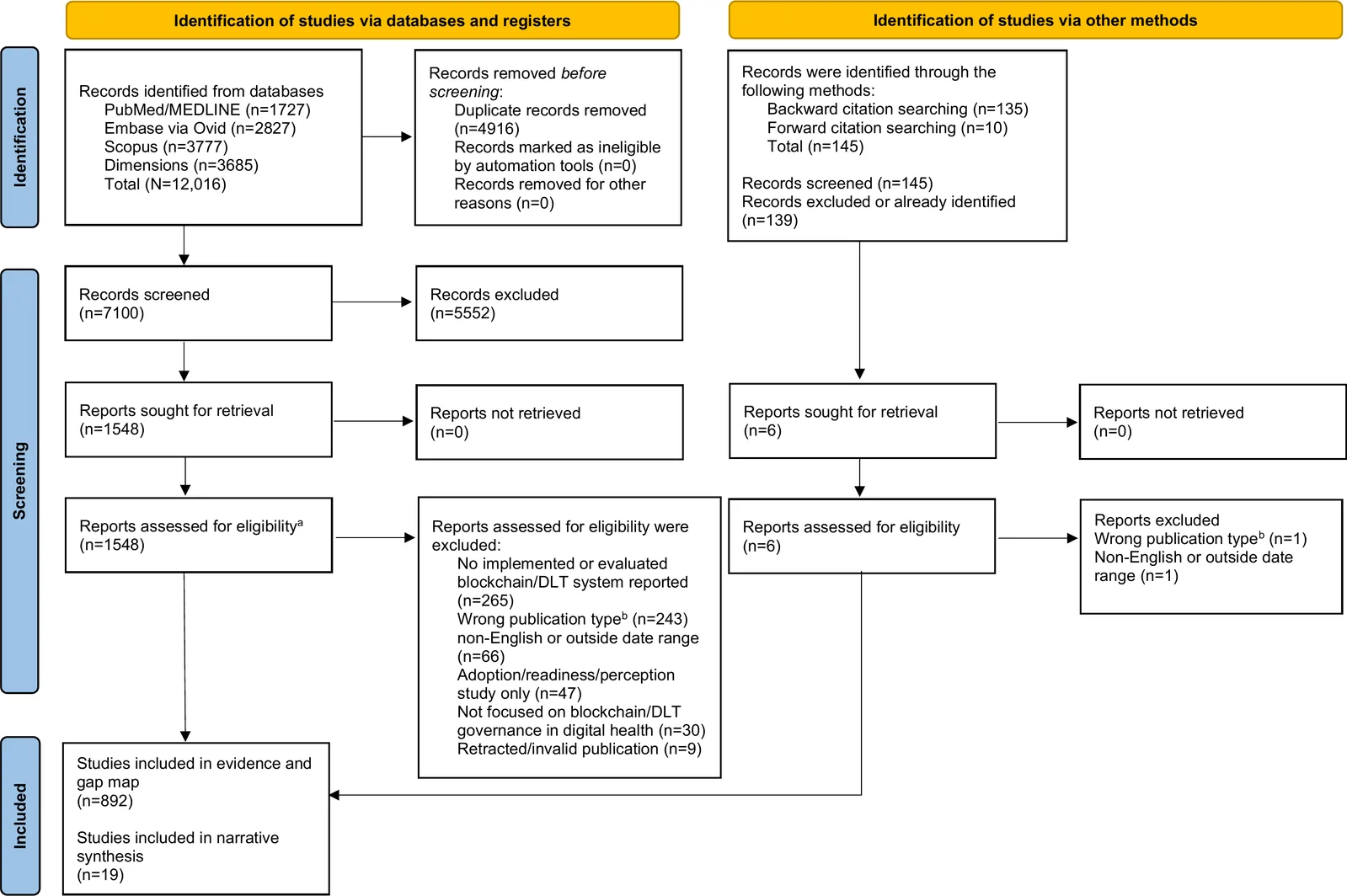
PRISMA (Preferred Reporting Items for Systematic Reviews and Meta-Analyses) 2020 flow diagram of the study selection for blockchain/distributed ledger technology (DLT) systems in digital health governance. ^a^Studies that reported an implemented artifact, technical evaluation, pilot/usability assessment, or operational deployment were included. ^b^The wrong publication type category included reviews, editorials, commentaries, opinion pieces, letters, protocols, conference abstracts without sufficient methodological detail, and other nonprimary research outputs.

### Evidence Gap Map: Application Domains, Governance Functions, and Evidence Maturity

The evidence base remained concentrated in early-stage and technical-evaluation studies. Overall, 418 studies were categorized as proof-of-concept/prototype studies, and 455 as simulated/benchmarked evaluations. Eighteen studies reported pilot/usability-tested implementation, and 1 reported an operational/real-world deployment (Figure 2A).

Studies were distributed across 6 health care application domains: EHR management and health information exchange (n=372), telemedicine/distributed care/Internet of Things (IoT)–enabled remote monitoring (n=250), clinical decision support/smart health care (n=82), public health surveillance/certification (n=72), clinical trials/research governance (n=67), and health data marketplace/monetization (n=49; Figure 2A).

Across domains, pilot/usability-tested implementation or operational/real-world deployment evidence remained limited and unevenly distributed. EHR management/health information exchange included 6 pilot/usability-tested implementation studies. Telemedicine/distributed care/IoT-enabled remote monitoring included 1 pilot/usability-tested implementation study and 1 operational/real-world deployment study. Clinical trials/research governance included 9 pilot/usability-tested implementation studies. Health data marketplace/monetization and clinical decision support/smart health care each included 1 pilot/usability-tested implementation study. No public health surveillance/certification studies were classified as higher maturity. Blockchain/DLT systems had, therefore, been proposed and technically evaluated across all 6 domains, but implementation evidence remained scarce.

Governance functions were coded as nonmutually exclusive because individual studies often addressed more than 1 governance mechanism. Across the included studies, 3273 governance-function codes were identified. The most frequent functions were privacy/security (n=750), interoperability/data sharing (n=679), access control (n=420), data/model integrity (n=386), and identity/authentication (n=349). Less frequent functions included auditability (n=224), AI/data governance (n=135), provenance/traceability (n=103), accountability/compliance (n=100), consent management (n=68), and incentives/value exchange (n=59). Governance-function codes were concentrated in proof-of-concept/prototype studies and simulated/benchmarked evaluations, with substantially fewer codes identified in pilot/usability-tested implementation or operational/real-world deployment studies (Figure 2B).

**Figure 2.**
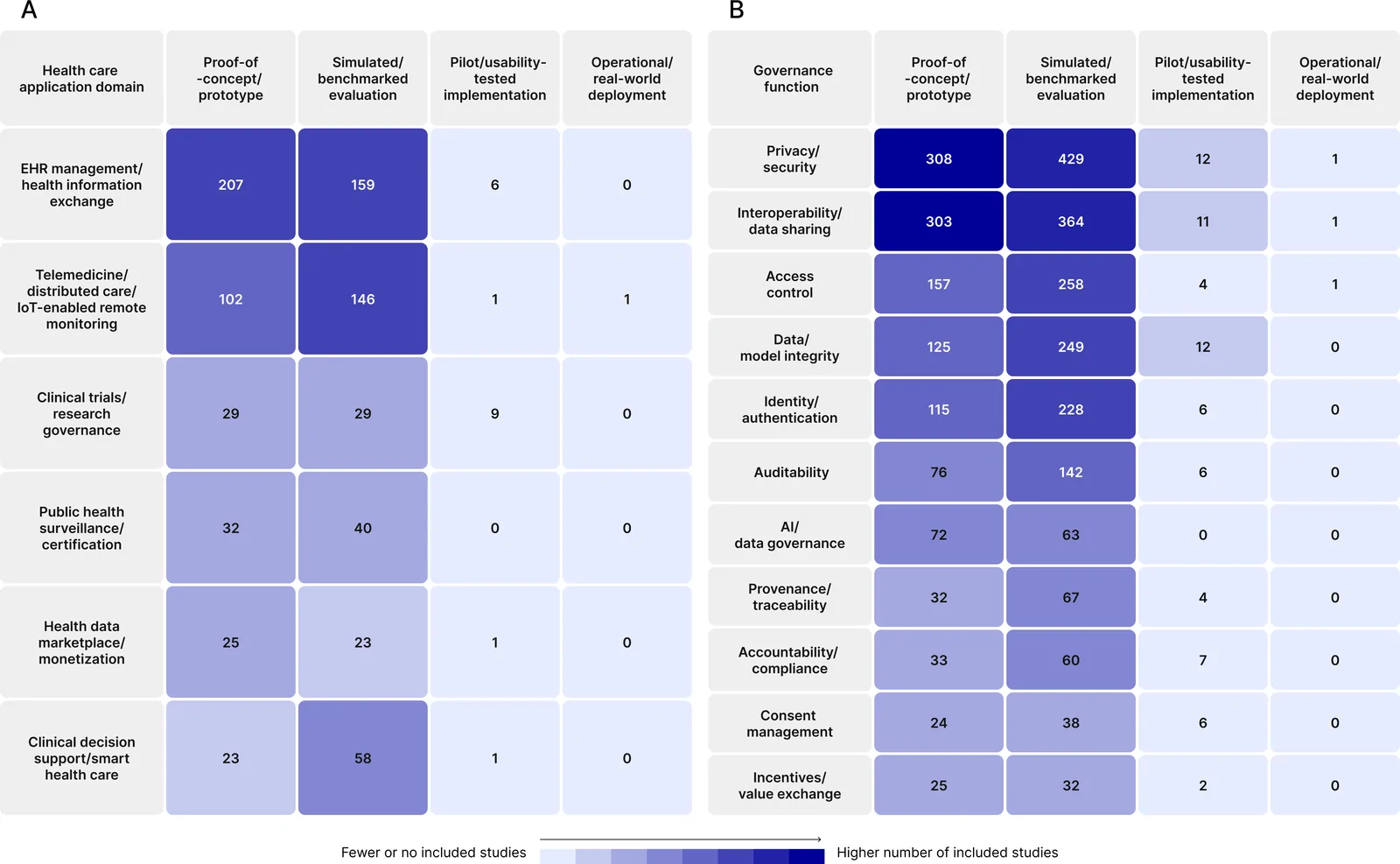
Evidence maturity of blockchain/distributed ledger technology studies by health care application domain (panel A) and governance function (panel B). Cell values indicate the number of included studies. Evidence maturity was categorized as proof-of-concept/prototype, simulated/benchmarked evaluation, pilot/usability-tested implementation, or operational/real-world deployment*.* EHR: electronic health record; IoT: Internet of Things.

### Governance Functions Across Health Care Application Domains

Governance functions were most frequently mapped to EHR management/health information exchange and telemedicine/distributed care/IoT-enabled remote monitoring (Figure 3). Across domains, privacy/security and interoperability/data sharing were the most widely represented functions, while the distribution of other functions varied by application domain.

**Figure 3.**
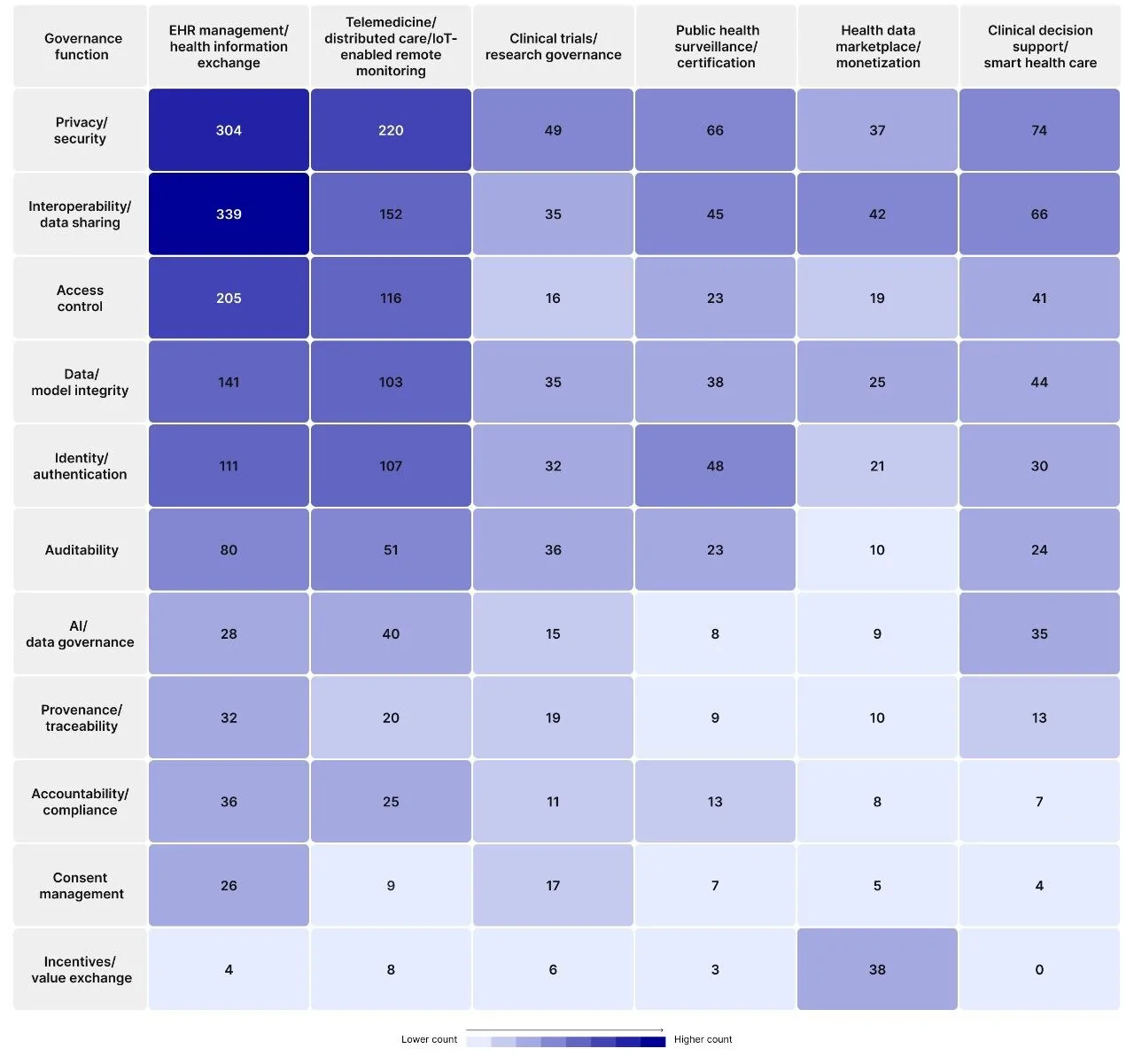
Distribution of blockchain-enabled governance functions across health care application domains. Cell values indicate the number of governance-function codes within each health care application domain. Governance functions were coded as nonmutually exclusive; individual studies could contribute to more than 1 function*.* EHR: electronic health record; IoT: Internet of Things.

### Temporal Distribution of the Evidence Base

Publication activity increased from 2017, with the highest annual volumes observed in 2022 and 2025, while the distribution of evidence maturity remained broadly similar over time. Proof-of-concept/prototype studies and simulated/benchmarked evaluations predominated across publication years, whereas pilot/usability-tested implementation studies remained uncommon and operational/real-world deployment was rare (Figure 4).

**Figure 4.**
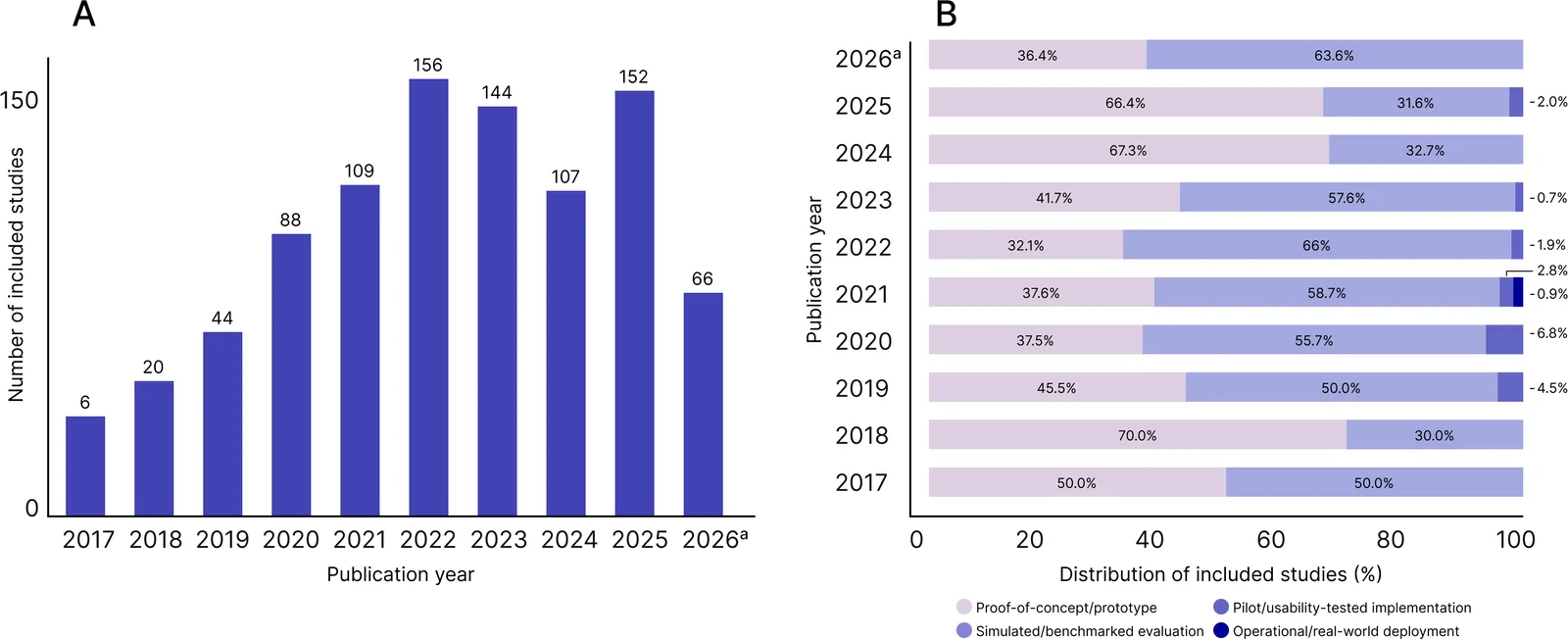
Temporal distribution of included blockchain/distributed ledger technology studies by publication volume (panel A) and evidence maturity (panel B). ^a^2026 includes only publications through February 28, 2026.

The temporal distribution of governance functions showed that privacy/security and interoperability/data sharing remained prominent across publication years, while the representation of other governance functions, including AI/data governance, accountability/compliance, consent management, and provenance/traceability, varied over time (Figure 5).

**Figure 5.**
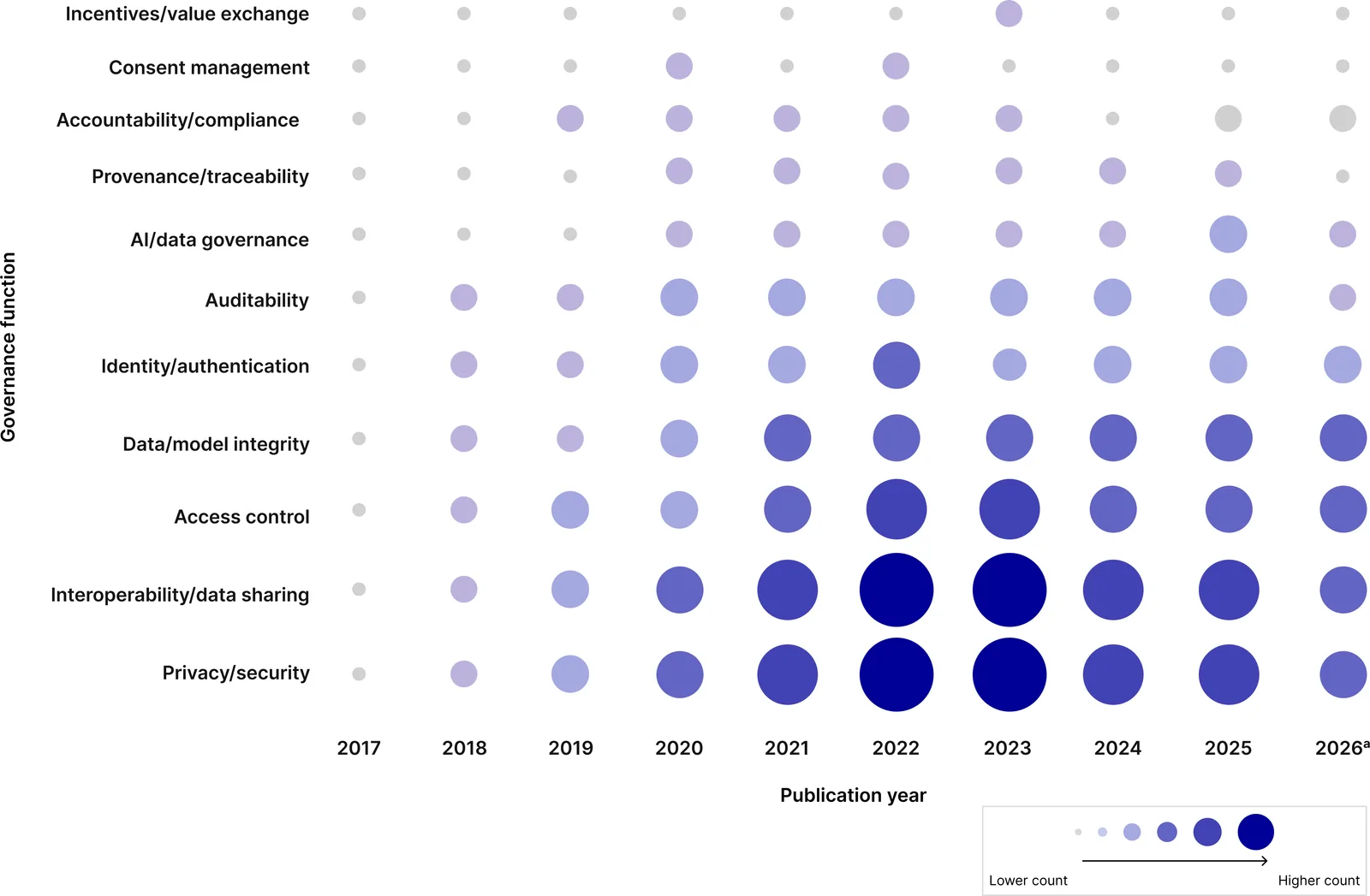
Temporal distribution of governance functions addressed by blockchain/distributed ledger technology studies. ^a^2026 includes only publications through February 28, 2026. Governance functions were nonmutually exclusive; individual studies could contribute to more than 1 function. Bubble size and color intensity indicate the number of governance-function codes within each publication year.

### Domain-Level Synthesis of Governance Mechanisms

Across health care application domains, blockchain/DLT systems were primarily positioned as infrastructure for coordinating and verifying data-related governance processes among distributed actors, with more specific roles varying by context. EHR/health information exchange studies focused on fragmented records, interoperability, and access permissions; telemedicine/distributed care/IoT-enabled remote monitoring studies emphasized identity, authentication, and secure patient-generated data flows; clinical trials/research governance studies addressed transparency, consent, and auditability; public health surveillance/certification studies focused on verification, certification, and cross-institutional trust; clinical decision support/smart health care studies emphasized provenance, data integrity, and accountability in AI-enabled workflows; and health data marketplace/monetization studies focused on patient control, consent, and value distribution.

Across domains, blockchain/DLT systems were mostly used as a coordination and verification layer rather than repositories for identifiable clinical data. Recurring mechanisms included access-control logging, consent recording, identity/authentication, auditability, provenance tracking, integrity verification, and interoperability support. Table 1 summarizes the main governance challenges, blockchain-enabled mechanisms, evidence maturity, and remaining limitations across the 6 health care application domains.

**Table 1.** Domain-level synthesis of governance challenges, blockchain-enabled mechanisms, and evidence maturity across digital health application domains^a^.

Domain	Key governance challenges	Blockchain-enabled governance mechanisms	Evidence maturity and limitations
EHR^b^ management/health information exchange (n=372)	Fragmented records; limited interoperability; insecure data exchange; unclear access permissions across institutions	Permissioned data exchange; access-control logs; patient-mediated sharing; on-chain hashes or transaction records; off-chain EHR storage	Largest domain; dominated by proof-of-concept/prototype and simulated/benchmarked evaluation studies; limited pilot/usability-tested implementation or operational/real-world deployment evidence; persistent integration challenges with existing EHR infrastructure
Telemedicine/distributed care/IoT^c^-enabled remote monitoring (n=250)	Remote identity verification; device authentication; insecure patient-generated data flows; fragmented patient-provider interactions	Decentralized identity; secure remote data exchange; authentication of patients, providers, and devices; smart-contract access permissions	Substantial technical evidence, mainly proof-of-concept/prototype and simulated/benchmarked evaluation studies; limited pilot/usability-tested implementation and operational/real-world deployment evidence; barriers in scalability, latency, and interoperability
Clinical decision support/smart health care (n=82)	Data provenance for AI/ML^d^; secure model training; accountability for data/model use; fragmented data pipelines	Blockchain-enabled federated learning coordination; audit trails for data/model processes; data integrity verification; smart-contract access control	Emerging and technically focused domain; mostly simulated/benchmarked studies; very limited pilot/usability-tested evidence; blockchain’s role in addressing model bias, data quality, or clinical effectiveness remains unevaluated
Public health surveillance/certification (n=72)	Verification of test or vaccination records; public-health data integrity; privacy-preserving certification; cross-border or cross-institutional trust	Verifiable credentials; tamper-evident certificates; identity/authentication systems; privacy-preserving data exchange; audit trails	Evidence concentrated in proof-of-concept/prototype and simulated/benchmarked evaluation studies; no confirmed pilot/usability-tested implementation or operational/real-world deployment evidence as a primary domain; implementation depends heavily on legal, institutional, and public-trust conditions
Clinical trials/research governance (n=67)	Trial transparency; protocol adherence; consent management; data integrity; auditability across multicenter research	Smart contracts for protocol rules; dynamic consent platforms; immutable audit trails; time-stamped records of research events and data access	Smaller but governance-relevant domain; comparatively higher concentration of pilot/usability-tested implementation studies; limited integration into routine trial infrastructure; regulatory and institutional adoption remain uncertain
Health data marketplace/monetization (n=49)	Patient control over secondary data use; transparency of data transactions; value distribution; consent tracking; risk of commodification	Tokenized data exchange; smart-contract payments or permissions; decentralized marketplaces; consent tracking; incentive mechanisms	Smallest domain; mostly proof-of-concept/prototype and simulated/benchmarked evaluation studies; limited pilot/usability-tested implementation evidence; no operational/real-world deployment; ethical, regulatory, and equity concerns remain unresolved

a Domains are ordered by the number of included studies (n).

b EHR: electronic health record.

c IoT: Internet of Things.

d ML: machine learning.

### Characteristics and Focused Synthesis of Higher-Maturity Studies

Table 2 summarizes the 19 studies classified as pilot/usability-tested implementation or operational/real-world deployment and included in the focused narrative synthesis. These studies varied in setting, sample size, duration, evaluation focus, and implementation status and were unevenly distributed across application domains. Full study-level extraction details are provided in Multimedia Appendix 4.

**Table 2.** Summary of pilot/usability-tested and operational blockchain/distributed ledger technology studies included in the focused narrative synthesis.

Primary domain	Higher-maturity studies^a^	Main evaluation focus	Blockchain/DLT^b^ governance role	Main implementation limitations
EHR^c^ management/health information exchange (n=6)	PatientDataChain (Setrio Soft SRL, Modex Ltd) [27]; iWellChain (Taipei Medical University Hospital) [28]; Homeless data platform (Dell Medical School, The University of Texas at Austin) [29]; PAGR^d^ e-prescribing (EirSystem Inc) [30]; MediLinker (The University of Texas at Austin) [31]; HealthPocket (Seoul National University, MediBloc Inc) [32]	System functionality, access logs, feasibility, e-prescribing workflow, usability, and patient-mediated information exchange	Permissioned data sharing, access logging, identity/credential management, consent or authorization recording, and integrity verification	Mostly small pilots or usability studies; limited sites, short follow-up, partial uptake, EHR integration challenges, scalability concerns, and limited evidence of sustained routine use
Telemedicine/distributed care/IoT^e^-enabled remote monitoring (n=2)	Dovetail digital consent (Dovetail Digital Ltd) [33]; IoHCS^f^/NUMED System (Naresuan University, 2nd Health Region, Ministry of Public Health) [34]	Patient/staff perceptions, staff satisfaction, system performance, and secure remote access	Consent traceability, secure patient-data access, identity and permission control, and cross-provider data availability	Limited independent evaluation of blockchain contribution; scalability, interoperability, technical reliability, and workflow integration remain uncertain
Clinical decision support/smart health care (n=1)	BRCA-CN consortium blockchain (National Institutes for Food and Drug Control [NIFDC]) [35]	Cross-institutional BRCA^g^ variant interpretation, interlaboratory concordance, VUS^h^-rate reduction, reporting time, expert consensus, and system performance	Multi-institutional consensus coordination, provenance, audit trails, data-sovereignty support, and accountability across laboratories	Limited to 6 laboratories and a defined deployment period; broader geographic representativeness, long-term sustainability, and full implementation of advanced privacy features remain uncertain
Public health surveillance/certification (n=0)	No higher-maturity studies identified	Not applicable	Proposed functions in the wider evidence map included certification, verification, privacy-preserving credentials, and surveillance-data integrity	No higher-maturity studies identified; limited evidence of testing with real users, public-health workflows, or health authorities
Clinical trials/research governance (n=9)	SUSMED mobile health (SUSMED Inc) [36]; SUSMED regulatory sandbox [37]; METORY platform (Jeonbuk National University Hospital and Seoul National University Hospital, Linux Foundation) [38]; METORY dynamic-consent trial [39]; Boehringer/IBM consent pilot (Boehringer Ingelheim, IBM, Linux Foundation) [40]; MediBloc change-monitoring app (Yonsei University College of Medicine and MediBloc Inc)[41]; Cancer Gene Trust (University of California) [42]; decentralized biobanking app (de-bi, co) [43]; MyHealthData platform (Seoul National University Hospital, MediBloc) [44]	Data integrity, fraud/tampering detection, electronic consent, protocol amendments, participant engagement, usability, monitoring time/cost, data sharing, and research transparency	Consent/event recording, immutable audit trails, hash-based integrity verification, provenance tracking, participant-facing transparency, and research-data accountability	Mostly pilot/feasibility studies; limited samples; short follow-up; uncertain regulatory acceptance; limited long-term integration into trial infrastructure; unresolved usability/comprehension issues
Health data marketplace/monetization (n=1)	Decentralized biobanking operational-feasibility case study [45]	Donor engagement, biospecimen tracking, participant engagement, app uptake, and blockchain-enabled access to specimen information	Transparency, donor recognition, provenance, participant engagement, and foundations for future incentive or data-governance models	Real-world feasibility evidence only; sustainability, governance, workflow integration, communications, ethical oversight, and scalability remain unresolved

a Developer/supplier information and blockchain platforms are reported as specified in the cited studies; underlying blockchain technologies and technical dependencies are not exhaustively detailed.

b DLT: distributed ledger technology*.*

c EHR: electronic health record.

d PAGR: prescription abuse greatly reduced.

e IoT: Internet of Things.

f IoHCS: Internet-of-Healthcare System.

g BRCA: breast cancer susceptibility gene.

h VUS: variant of uncertain significance.

### EHR Management/Health Information Exchange

Six higher-maturity studies were categorized as EHR management/health information exchange [27-32]. These studies examined blockchain/DLT systems for personal health records, referral data exchange, identity and document sharing, electronic prescribing, decentralized identifiers, verifiable credentials, and patient-mediated health information exchange. PatientDataChain and iWellChain were evaluated in clinical settings and used off-chain storage with blockchain-based mechanisms to support data integrity, permissions, and patient-authorized access [27,28]. Other studies examined identity and document portability for people experiencing homelessness [29], blockchain-supported electronic prescribing in family medicine clinics [30], decentralized identity and credential sharing through MediLinker, a blockchain-based decentralized health information management platform [31], and Fast Healthcare Interoperability Resources (FHIR)–based patient-mediated information exchange through HealthPocket, a blockchain-based mobile health information exchange platform [32].

Across these studies, blockchain/DLT systems supported permissioned access, patient authorization, identity management, data integrity, auditability, and interoperability. However, implementation remained limited to pilots, usability studies, or early clinical demonstrations, with short follow-up, restricted settings, partial uptake, and unresolved integration challenges with existing EHR infrastructure (Multimedia Appendix 4).

### Telemedicine/Distributed Care/IoT-Enabled Remote Monitoring

Two higher-maturity studies were categorized in the telemedicine, distributed care, and IoT-enabled remote monitoring domain [33,34]. Dovetail, a blockchain-based digital consent mobile application for patients with diabetes, was evaluated in a UK general practice, focusing on patient-managed consent for data sharing between the practice and diabetes self-management tools [33]. The Internet-of-Healthcare System (IoHCS), a blockchain-supported health information network implemented across more than 350 hospitals, provided secure patient access in Thailand, with staff feedback reporting high satisfaction with usability, installation, maintenance, and security [34].

These studies used blockchain/DLT systems to support consent management, secure remote access, identity and permission control, data integrity, and cross-provider data availability. Evidence remained limited to 1 digital consent pilot and 1 operational health information access network, with uncertainty around scalability, interoperability, technical reliability, and integration into clinical workflows.

### Clinical Trials/Research Governance

Nine higher-maturity studies examined applications of blockchain/DLT systems in clinical trials/research governance [36-44]. These studies addressed consent management, participant engagement, data integrity, auditability, data validation, protocol amendments, and research-data sharing. Motohashi et al [36] applied cryptographic hash chains in an insomnia clinical trial and demonstrated detection of simulated fraudulent access. Hirano et al [37] evaluated blockchain-based data validation in a breast cancer clinical trial conducted within a Japanese regulatory sandbox.

Other studies focused on participant-facing governance. METORY was developed as a blockchain-based dynamic consent platform [38] and subsequently evaluated in a decentralized multicenter trial involving 60 participants [39]. Mak et al [40] piloted a Hyperledger Fabric system for informed consent and patient engagement among 12 patients with moderate to severe psoriasis. Additional applications included monitoring changes to medical records [41], authenticated sharing of deidentified genomic and clinical cancer data [42], decentralized biobanking [43], and patient-facing access to health records and research eligibility information [44].

Across these studies, blockchain/DLT systems were used less as repositories for research data than as accountability layers for recording consent, preserving data integrity, validating submissions, tracking access or amendments, and supporting transparent participant engagement. Most studies remained small-scale pilots, usability studies, or early demonstrations, with limited evidence of long-term integration into trial-management systems, regulatory workflows, ethics-review processes, or routine research infrastructure.

### Public Health Surveillance and Certification

No higher-maturity studies were categorized as public health surveillance or certification. The wider evidence gap map included studies on vaccination and testing certificates, outbreak surveillance, public-health data integrity, cross-border verification, and privacy-preserving credentials. However, these remained at the proof-of-concept/prototype or simulated/benchmarked evaluation stage. Evidence of testing with real users, implementation in public-health workflows, or adoption across health authorities was limited.

### Clinical Decision Support/Smart Health Care/AI-Enabled Data Governance

One higher-maturity study was categorized in the clinical decision support, smart health care, and AI-enabled data governance domains [35]. The breast cancer susceptibility gene-China (BRCA-CN) study deployed a consortium blockchain framework for cross-institutional breast cancer susceptibility gene variant interpretation in the Chinese population. Six clinical genetics laboratories shared and curated variant evidence through a permissioned blockchain network incorporating smart-contract governance, expert consensus review, audit trails, and AI-supported variant assessment [35]. The study positioned blockchain/DLT as governance infrastructure for standardizing data contribution, preserving provenance, coordinating expert review, and supporting cross-institutional accountability. However, evidence in this domain remained limited to a single deployment, and blockchain did not resolve broader challenges involving model validity, bias, data quality, clinical effectiveness, or routine integration.

### Health Data Marketplace, Monetization, and Incentives

One higher-maturity study was categorized in the health data marketplace/monetization domain [45]. This mixed methods case study evaluated the operational feasibility of a decentralized biobanking application at a US academic medical center. The study recruited 1080 biobank members over 10 weeks; 405 downloaded the application, and 140 tested the blockchain component. Of these, 89.3% (125/140) successfully claimed a nonfungible token representing their connection to donated biospecimens and access to related specimen information [45].

Rather than functioning as a conventional data marketplace, the platform used blockchain to support transparency, donor recognition, participant engagement, and future incentive and governance models. The study suggests that blockchain/DLT systems may support patient-facing transparency and value recognition in biobanking; however, sustainability, workflow integration, communication, scalability, and ethical oversight remain unresolved [45].

## Discussion

### Principal Findings and Contribution

This study aimed to characterize how blockchain/DLT systems have been applied to health-data governance, identify the application domains and governance functions addressed, and assess the maturity of the supporting evidence. Collectively, the findings reveal 3 key insights. First, the literature is expansive but concentrated at low maturity: only 19 of 892 studies reported a pilot, usability-tested, or operational deployment, and a single study was classified as an operational/real-world deployment. Second, blockchain/DLT systems were rarely positioned as repositories for identifiable clinical data or as direct clinical interventions. Instead, they functioned as governance-layer infrastructure intended to coordinate access, verify transactions, record permissions, preserve audit trails, and support trust across distributed actors. Third, higher-maturity evidence was unevenly distributed across domains. EHR/health information exchange, clinical trials/research governance, and telemedicine/distributed care accounted for most applied studies, whereas public health surveillance/certification, health data marketplace/monetization, and clinical decision support/smart health care were dominated by early-stage technical work.

Previous reviews have mapped blockchain/DLT applications across health care but have less often examined their governance role or the maturity of the supporting evidence [15,24]. This review maps application domains, governance functions, and evidence maturity together, showing that the scale of the literature risks overstating the field’s progress. In other words, many systems have been prototyped, simulated, or benchmarked, but few have been evaluated in routine settings [20,24,46]. This is consistent with Yeung et al [46] findings that health care blockchain applications had shown little real transformative deployment and 5 years on, the gap persists.

Beyond validating that gap, this review reveals 2 novel contributions. By coding governance function and evidence maturity together, this study shows that some of the field’s most frequently reported strengths, privacy/security, interoperability/data sharing, and access control are concentrated where the evidence is least mature: in proof-of-concept and simulated/benchmarked evaluation studies rather than implemented or deployed systems. Additionally, this study demonstrates that the specific contribution of DLT properties to measured outcomes could generally not be isolated from that of interface design, system integration, and conventional infrastructure [27,28,31,32,37-45]. In line with this, systems in which the ledger was most central did not reach sustained use [27,28,31,32,37-45]. We state this as an observed pattern, not a causal relationship as the evidence is based on limited studies, several of which evaluate the same or closely related architecture, and is confined to indexed literature that may underrepresent operational deployments.

### Blockchain/DLT Systems as Governance-Layer Infrastructure

The recurring mechanisms were similar across domains: access-control logs, consent and authorization records, identity and authentication credentials, provenance records, integrity checks, audit trails, and transaction histories [47-51]. In each case, identifiable health data remained off-chain, while the ledger held only hashes, metadata, permissions, or transactions to support verification and accountability [48-50]. This locates blockchain’s contribution not in replacing existing health information systems but in providing a coordination and verification layer across fragmented data environments [8,20]. Blockchain/DLT systems may therefore be most relevant where governance itself is distributed and where multiple organizations, professionals, patients, researchers, regulators, or commercial actors must exchange, authorize, validate, or audit data without relying on a single central authority [15,52]. Such settings include cross-institutional health information exchange [18,51], multicenter clinical trials [38-42], decentralized research platforms [22,52], genomic databases [21,35,42], biobanks [43,45], and patient-mediated data-sharing systems [31,33,53].

### From Technical Demonstration to Implementation

A central finding is the gap between technical possibility and implemented governance. A technically functional system is not necessarily a usable, trusted, sustainable, or governable intervention [15,20,24,46]. The higher-maturity studies suggest that blockchain/DLT systems may support consent management, auditability, data validation, patient-facing transparency, and cross-institutional coordination. These functions are particularly relevant to clinical trials and research governance, where transparency, protocol adherence, and accountability remain persistent concerns [54,55].

Notably, however, the measured benefits across these studies typically arose from interface design, system integration, remote monitoring, or conventional data infrastructure rather than from distributed-ledger properties themselves [27-45], and none of the included higher-maturity studies reported evidence of sustained routine use beyond the evaluation period [27-45]. Where blockchain/DLT contributed, it did so by producing more traceable records of consent, amendments, access, and trial events when embedded within appropriate regulatory and institutional frameworks, not by independently delivering the measured outcome [52,56]. Implementation is less dependent on the ledger than on institutional participation, EHR integration, governance authority, user trust, workflow fit, legal accountability, cost, and long-term maintenance [57,58].

The value of blockchain/DLT systems is therefore highly context-dependent. Their use is most justified where governance involves multiple institutions, low baseline trust, distributed authority, or audit requirements that must be met without a single central actor, and blockchain/DLT systems should not be treated as default solutions [46]. In centralized or highly integrated systems, similar objectives are often achievable through conventional databases, federated architectures, access-control systems, secure audit logs, or trusted institutional governance, without the added complexity of DLT infrastructure [15,20,51]. Reviews and implementation studies consistently identify constraints around scalability, latency, throughput, storage design, interoperability, cost, technical complexity, standardization, and regulatory uncertainty [18,57,58]. Recent systematic reviews continue to identify the same implementation barriers despite the growing literature, suggesting that many of the obstacles reported several years ago remain unresolved [59,60]. These constraints are especially consequential in clinical environments, where added workflow burden, delay, or fragmentation can undermine adoption.

Even where they support verification and traceability, blockchain/DLT systems do not resolve the broader governance challenges of digital health. Legal accountability, regulatory compliance, data stewardship, and ethical oversight remain external to the technology: a ledger can record that consent was given, amended, or withdrawn, but cannot ensure that consent was informed or that power imbalances between patients, institutions, and commercial actors have been addressed [53]. This is particularly important in health data marketplaces, incentive models, and AI-enabled health care. Incentives alone may be insufficient to promote sensitive data sharing while privacy, security, trust, and regulatory concerns remain unresolved [61]. Marketplace prototypes remain constrained by unresolved questions of governance, regulation, interoperability, data quality, ownership, and compensation standards [62,63]. Governance frameworks must therefore be designed around the full sociotechnical system, not the ledger alone.

### Implications for Policy, Implementation, and Future Research

These findings suggest blockchain/DLT systems should be evaluated as components of broader governance frameworks rather than stand-alone innovations. For policymakers and health-system leaders, the relevant question is not whether blockchain is technically novel but whether it improves governance performance in measurable ways, consent traceability, transparency, accountability, access-control reliability, interoperability, administrative efficiency, user trust, cost-effectiveness, and sustainability [7,24]. Future research should move beyond proof-of-concept and simulated/benchmarked evaluation studies toward implementation-focused evaluation, reporting adoption, usability, integration with existing systems, governance roles, data-protection compliance, costs, unintended consequences, and long-term maintenance. Recent reviews likewise emphasize the need for more empirical evaluations, real-world deployments, and human-centered implementation studies rather than additional conceptual architectures [59,60]. Comparative studies are needed to establish when blockchain/DLT systems offer genuine advantages over conventional alternatives. For emerging areas such as data marketplaces, incentive models, and AI-enabled governance, future work should address ethical and regulatory questions directly, including data commodification, equity, consent withdrawal, and accountability for secondary use [52,53,61].

### Limitations

Several limitations should be acknowledged. First, evidence was restricted to peer-reviewed English-language publications. Relevant implementation activity may appear in gray literature, policy documents, industry reports, white papers, or regulatory submissions, particularly in fast-moving areas such as data marketplaces and public health certification. Second, the evidence base was heterogeneous across domains, technologies, governance functions, and evaluation methods, limiting comparability. Third, no formal risk-of-bias or quality appraisal was conducted, consistent with scoping review methodology [25]; evidence maturity should not be interpreted as equivalent to methodological quality or effectiveness. Fourth, classifying domains, governance functions, and maturity required interpretive judgment. Although coding categories were defined and applied consistently, some studies spanned multiple domains, and the primary-domain classification may have simplified cross-domain studies.

In conclusion, this study identified that blockchain/DLT systems are most consistently positioned as governance-layer infrastructure for recording, verifying, and coordinating data-related actions across distributed actors. Their most active role is currently to support coordination, verification, consent traceability, auditability, and accountable data exchange within complex digital health ecosystems, rather than replacing clinical systems or storing identifiable health data on-chain. The broader implication is that blockchain/DLT systems should be judged by their contribution to governance performance rather than by technological novelty.

For health care systems, regulators, researchers, and patients, the relevant question is whether blockchain-enabled systems can make data access more accountable, consent more transparent, data exchange more trustworthy, and distributed collaboration more reliable. To our knowledge, peer-reviewed evidence supporting these outcomes remains limited, and where higher-maturity systems reported benefits, the specific contribution of the distributed ledger was often difficult to isolate. Future work should move beyond technical demonstration and test whether blockchain/DLT systems can be integrated, sustained, and shown to improve accountability, consent traceability, and trusted data exchange in mature, deployed systems.

## Supplementary material

10.2196/96098 Multimedia Appendix 1 Search strategies used in database searches.

10.2196/96098 Multimedia Appendix 2 Summary of database search strategy.

10.2196/96098 Multimedia Appendix 3 Complete list of included studies, health care application domains, governance functions, and evidence maturity levels.

10.2196/96098 Multimedia Appendix 4 Study-level characteristics, evaluation focus, blockchain/distributed ledger technology governance role, and implementation limitations of the 19 higher-maturity studies included in the focused narrative synthesis.

10.2196/96098 Checklist 1 PRISMA-ScR fillable checklist.

## Abbreviations

DLT: distributed ledger technology
EHDS: European Health Data Space
EHR: electronic health record
FHIR: Fast Healthcare Interoperability Resources
IoHCS: Internet-of-Healthcare System
IoT: Internet of Things
OECD: Organisation for Economic Co-operation and Development
PRISMA: Preferred Reporting Items for Systematic Reviews and Meta-Analyses
PRISMA-S: Preferred Reporting Items for Systematic Reviews and Meta-Analyses literature search extension
PRISMA-ScR: Preferred Reporting Items for Systematic Reviews and Meta-Analyses extension for Scoping Reviews
