# Supplementary material for: Blockchain for Digital Health Governance: Evidence Gap Map and Scoping Review
Source: J Med Internet Res. 2026 Oct 7;28:e96098. doi: 10.2196/96098 (PMC13645320; doi:10.2196/96098)
Supplement: Supplementary file 2 [file jmir-v28-e96098-s001.docx]

**Multimedia Appendix 1.** Search strategies used in database searches

| Search database | Search string | Records retrieved |
| --- | --- | --- |
| PubMed/MEDLINE | (  "Blockchain"[Mesh]  OR blockchain[tiab]  OR "block chain"[tiab]  OR "distributed ledger"[tiab]  OR "distributed ledger technology"[tiab]  OR DLT[tiab]  OR "smart contract*"[tiab]  OR "decentralized identit*"[tiab]  OR "self-sovereign identit*"[tiab]  OR tokeni*[tiab]  OR "non-fungible token*"[tiab]  OR NFT[tiab]  OR Web3[tiab]  )  AND  (  "Delivery of Health Care"[Mesh]  OR "Health Information Management"[Mesh]  OR "Electronic Health Records"[Mesh]  OR "Telemedicine"[Mesh]  OR "Artificial Intelligence"[Mesh]  OR "Clinical Trials as Topic"[Mesh]  OR "Biomedical Research"[Mesh]  OR health[tiab]  OR healthcare[tiab]  OR "health care"[tiab]  OR medical[tiab]  OR clinical[tiab]  OR "digital health"[tiab]  OR eHealth[tiab]  OR mHealth[tiab]  OR telemedicine[tiab]  OR telehealth[tiab]  OR "remote care"[tiab]  OR "electronic health record*"[tiab]  OR EHR[tiab]  OR "clinical trial*"[tiab]  OR "clinical research"[tiab]  OR "biomedical research"[tiab]  OR "artificial intelligence"[tiab]  OR AI[tiab]  OR "machine learning"[tiab]  OR "health data"[tiab]  OR "medical data"[tiab]  )  AND  (  "Informed Consent"[Mesh]  OR "Privacy"[Mesh]  OR "Computer Security"[Mesh]  OR "Access to Information"[Mesh]  OR "Health Information Interoperability"[Mesh]  OR governance[tiab]  OR "data governance"[tiab]  OR accountability[tiab]  OR consent[tiab]  OR "dynamic consent"[tiab]  OR "informed consent"[tiab]  OR "data sharing"[tiab]  OR "data exchange"[tiab]  OR "health information exchange"[tiab]  OR "access control"[tiab]  OR permission*[tiab]  OR audit*[tiab]  OR "audit trail*"[tiab]  OR auditability[tiab]  OR provenance[tiab]  OR traceability[tiab]  OR transparency[tiab]  OR interoperab*[tiab]  OR privacy[tiab]  OR security[tiab]  OR compliance[tiab]  OR regulatory[tiab]  OR regulation[tiab]  OR "data stewardship"[tiab]  OR "identity management"[tiab]  OR "data monetization"[tiab]  OR "data marketplace*"[tiab]  )  AND  ("2010/01/01"[Date - Publication] : "2026/02/28"[Date - Publication])  AND english[Language] | 1,727 |
| Scopus | TITLE-ABS-KEY ( blockchain OR "block chain" OR "distributed ledger" OR "distributed ledger technology" OR "smart contract*" ) AND TITLE-ABS-KEY ( "digital health" OR healthcare OR "health care" OR medical OR clinical OR telemedicine OR telehealth OR "electronic health record*" OR EHR OR "clinical trial*" OR "clinical research" OR "artificial intelligence" OR "machine learning" OR "health data" OR "medical data" ) AND TITLE-ABS-KEY ( "data governance" OR governance OR "dynamic consent" OR "informed consent" OR "data sharing" OR "data exchange" OR "health information exchange" OR "access control" OR "audit trail*" OR auditability OR provenance OR traceability OR interoperab* OR "regulatory compliance" OR "data stewardship" OR "identity management" OR "data monetization" OR "data marketplace*" ) AND PUBYEAR > 2009 AND PUBYEAR < 2027 AND LIMIT-TO (LANGUAGE, "English") | 3,777 |
| Embase via Ovid | 1. blockchain.ti,ab,kw. OR "block chain".ti,ab,kw. OR "distributed ledger".ti,ab,kw. OR "distributed ledger technology".ti,ab,kw. OR DLT.ti,ab,kw. OR "smart contract*".ti,ab,kw.  2. exp digital health/ OR exp telemedicine/ OR exp electronic health record/ OR exp artificial intelligence/ OR exp clinical trial/ OR exp biomedical research/ OR health.ti,ab,kw. OR healthcare.ti,ab,kw. OR "health care".ti,ab,kw. OR medical.ti,ab,kw. OR clinical.ti,ab,kw. OR "digital health".ti,ab,kw. OR eHealth.ti,ab,kw. OR mHealth.ti,ab,kw. OR telemedicine.ti,ab,kw. OR telehealth.ti,ab,kw. OR "remote care".ti,ab,kw. OR "electronic health record*".ti,ab,kw. OR EHR.ti,ab,kw. OR "clinical trial*".ti,ab,kw. OR "clinical research".ti,ab,kw. OR "biomedical research".ti,ab,kw. OR "artificial intelligence".ti,ab,kw. OR AI.ti,ab,kw. OR "machine learning".ti,ab,kw. OR "health data".ti,ab,kw. OR "medical data".ti,ab,kw.  3. exp data sharing/ OR exp informed consent/ OR exp privacy/ OR exp computer security/ OR exp interoperability/ OR governance.ti,ab,kw. OR "data governance".ti,ab,kw. OR accountability.ti,ab,kw. OR consent.ti,ab,kw. OR "dynamic consent".ti,ab,kw. OR "informed consent".ti,ab,kw. OR "data sharing".ti,ab,kw. OR "data exchange".ti,ab,kw. OR "health information exchange".ti,ab,kw. OR "access control".ti,ab,kw. OR permission*.ti,ab,kw. OR audit*.ti,ab,kw. OR "audit trail*".ti,ab,kw. OR auditability.ti,ab,kw. OR provenance.ti,ab,kw. OR traceability.ti,ab,kw. OR transparency.ti,ab,kw. OR interoperab*.ti,ab,kw. OR privacy.ti,ab,kw. OR security.ti,ab,kw. OR compliance.ti,ab,kw. OR regulatory.ti,ab,kw. OR regulation.ti,ab,kw. OR "data stewardship".ti,ab,kw. OR "identity management".ti,ab,kw. OR "data monetization".ti,ab,kw. OR "data marketplace*".ti,ab,kw.  4. 1 AND 2 AND 3  5. limit 4 to English language  6. limit 5 to yr="2010 - 2026" | 2,827 |
| Dimensions.ai | ("blockchain" OR "distributed ledger")  AND  ("digital health" OR healthcare OR "health care" OR medical OR clinical OR telemedicine OR telehealth OR "electronic health record" OR "clinical trial" OR "artificial intelligence" OR "health data")  AND  ("data governance" OR "dynamic consent" OR "data sharing" OR "access control" OR auditability OR provenance OR interoperability OR "regulatory compliance")  Filters:   - Publication type: article; - publication years: 2010-2026, - language: English | 3,685 |
