# Supplementary material for: Blockchain for Digital Health Governance: Evidence Gap Map and Scoping Review
Source: J Med Internet Res. 2026 Oct 7;28:e96098. doi: 10.2196/96098 (PMC13645320; doi:10.2196/96098)
Supplement: Supplementary file 3 [file jmir-v28-e96098-s002.docx]

**Multimedia Appendix 2.** Summary of database search strategy

| Database | Controlled vocabulary used | Free-text fields searched | Main concepts combined |
| --- | --- | --- | --- |
| PubMed/MEDLINE | MeSH terms | Title/Abstract | Blockchain/distributed ledger technologies  AND  digital health/healthcare contexts  AND  governance-related functions |
| Embase via Ovid | EMTREE terms | Title/Abstract/Keyword | Blockchain/distributed ledger technologies  AND  digital health/healthcare contexts  AND  governance-related functions |
| Scopus | Not applicable | Title/Abstract/Keyword | Blockchain/distributed ledger technologies  AND  digital health/healthcare contexts  AND  governance-related functions |
| Dimensions | Not applicable | Title/Abstract | Blockchain/distributed ledger technologies  AND  digital health/healthcare contexts  AND  governance-related functions |
