# Supplementary material for: Blockchain for Digital Health Governance: Evidence Gap Map and Scoping Review
Source: J Med Internet Res. 2026 Oct 7;28:e96098. doi: 10.2196/96098 (PMC13645320; doi:10.2196/96098)
Supplement: Supplementary file 4 [file jmir-v28-e96098-s004.docx]

**Multimedia Appendix 4.** Study-level characteristics, evaluation focus, blockchain/distributed ledger technology governance role, and implementation limitations of the 19 higher-maturity studies included in the focused narrative synthesis.

| Study | Setting/country | Sample/duration | Evaluation focus | Blockchain/DLT systems governance role | Implementation status and limitations |
| --- | --- | --- | --- | --- | --- |
| EHR/health information exchange | | | | | |
| PatientDataChain (1) | 1 private clinic, Bucharest, Romania | 100 patients; 1,144 transactions; 3 months | System functionality and transaction throughput/scalability; no patient or clinician outcomes and no usability scores reported. | Off-chain health data storage with blockchain-based integrity and permission mechanisms; ledger used to record/verify transactions rather than store clinical data. | Single clinical setting; no evidence of sustained routine implementation; scalability and EHR integration remained limitations. |
| iWellChain (2) | 1 teaching hospital and 4 clinics, Taiwan | 392 registered users; 3 months of access logs | Patient app access-log behavior, including record types accessed during referral-related use. | Consortium Ethereum used to record permissions and access events; health records stored off-chain/IPFS. | Deployed in real clinics but with partial uptake; older devices limited access; scalability and expansion beyond the participating clinics remained barriers. |
| Homeless data platform (3) | Austin, Texas, United States; Bloomberg Mayor's Challenge pilot | 2 people experiencing homelessness tested the platform; approximately 34 stakeholders engaged; 6-month project | Qualitative feasibility assessment of assumptions around identity and document sharing across health and social service providers. | Blockchain was proposed to support identity/document portability; the pilot raised questions about whether blockchain was necessary relative to existing systems. | Pilot did not progress to sustained implementation; barriers included usability, privacy/security, HIPAA ambiguity, governance, cost, and organizational readiness. |
| PAGR e-prescribing (4) | 3 family-medicine clinics, Tennessee, United States | 126 patients; 107 prescriptions; 3 months | Prescription-writing time, medication-reconciliation accuracy, and prescription drug monitoring program compliance. | Blockchain component used to record prescription-related transactions/audit information; main measured workflow effects were linked to prescribing-system integration and user interface functions. | Small vendor-supported pilot; no evidence of routine adoption; limitations included learner bias and absence of standard implementation metrics. |
| MediLinker (5) | University of Texas at Austin laboratory; simulated primary-care sessions | Synthetic patients, usability testers, and 913-person MTurk survey | Technical feasibility and willingness-to-use survey; no real-patient clinical deployment. | Self-sovereign identity and verifiable credentials using Hyperledger Indy/Aries; credentials remained off-ledger. | Phased research-and-development program; real-clinic deployment was planned for a later phase; limitations included deployment, device, scaling, trust, and legal issues. |
| HealthPocket (6) | 1 tertiary hospital and 1 primary clinic, South Korea | 116 users; at least 1 month; 8 users in the cross-institution referral case | Usability measures including perceived ease of use, usefulness, and satisfaction. | MediBloc Panacea ledger used for integrity verification; health information exchange used HL7 FHIR and off-chain data handling. | Pilot between two institutions; no evidence of sustained routine integration; limitations included low ease-of-use scores, limited sites, and lack of full EHR linkage. |
| ****Telemedicine/distributed care/IoT-enabled remote monitoring**** | | | | | |
| Dovetail digital consent (7) | 1 general practice, England; NHS SBRI pilot | 23 patients and 13 staff; perception survey/concept evaluation | Patient and staff attitudes toward digital consent compared with paper-based processes. | Blockchain-based consent ledger intended to support transparent, traceable, and revocable consent. | Concept/pilot evaluation rather than sustained use; network outages occurred and the independent contribution of blockchain was not isolated. |
| Internet-of-Healthcare (IoHCS / NUMED) (8) | Thailand; reported deployment across 350 hospitals | 8,578 patients; 221,269 blocks; staff satisfaction survey n=143 | Staff satisfaction and system performance benchmarks. | Blockchain was one component of a broader secure patient-data access architecture using mobile access, encryption, MQTT communication, and key-management infrastructure. | Operational use reported, but scalability beyond participating hospitals and security remained key concerns; the relative contribution of blockchain versus the wider system architecture was unclear. |
| Clinical trials/research governance | | | | | |
| SUSMED mHealth/insomnia (9) | Japan; insomnia CBT-I clinical trial | Real trial participants; sample size not clearly stated in the system-validation report | Security/attack-resistance testing, including detection of simulated fraudulent access; not a clinical, usability, or adoption outcome. | Blockchain and client hashchains used for tamper-resistant storage and data-integrity verification. | No usage-over-time or outcome follow-up reported; limitations included client-device compromise, 51% attack risk with few nodes, and untested scalability across simultaneous trials. |
| SUSMED breast-cancer, regulatory sandbox (10) | Japan; National Cancer Center Japan; breast-cancer survivor HIIT trial under regulatory sandbox | Real RCT participants; blockchain paper reports system behavior rather than trial outcomes | Data integrity and system resilience, including validation against tampering and reported zero downtime during an AWS Tokyo outage. | Blockchain/client-hashchain architecture used for integrity validation and regulatory-grade record keeping; resilience also depended on conventional cloud infrastructure. | Operational during the trial but no post-study sustainment data; limitations included untested scalability across varied trials and multi-device data capture. |
| METORY platform-development paper (11) | South Korea; Seoul National University Hospital and Jeonbuk National University Hospital | Preliminary survey of 61 investigators/personnel; UAT groups; 60 trial subjects in related multicenter study | Platform-development process, user acceptance testing, and usability/user-experience ratings. | Hyperledger Fabric used to anchor consent-related hashes and QR codes; consent documents stored off-chain in an encrypted external database. | Platform was used in the related trial but no operational continuation was reported; limitations included external database overhead, single-point-of-failure concerns, transaction failures, and limited implementation of some eConsent functions. |
| METORY decentralized dynamic-consent trial (12) | South Korea; two-center decentralized trial using virtual drugs | 60 subjects; 4 weeks | Consent rate, response time to amendments, drug adherence, and procedural adherence over time. | Hyperledger Fabric used to record consent and amendment events; the app supported notification and participant-facing consent workflows. | Feasibility evaluation only; no continuation reported; limitations included misunderstanding/ignoring platform information, no paper-consent comparator, and limited adverse-event realism with virtual drugs. |
| Boehringer/IBM psoriasis consent pilot (13) | Canada; 5 of 8 sites in a global phase II psoriasis trial; Hyperledger Fabric v1.4 | 12 pilot participants; 7 study coordinators; 3 clinical research associates; March-November 2019 | Monitoring visit time and cost, non-compliances, and user-reported trust/transparency/empowerment. | Blockchain used to create an immutable consent/status record and enable remote monitoring workflows. | Pilot only; blockchain records were not accepted by health authorities at the time; limitations included small sample size, limited compliance comparisons, mixed coordinator/CRA perceptions, and scalability/interoperability concerns. |
| MediBloc medical-record-change monitoring app (14) | South Korea; two primary-care pediatric clinics | 70 enrolled; 44 analyzed; approximately 34-day use window; SUS survey | Usability and usage patterns; no actual document changes occurred during the study. | MediBloc Panacea ledger designed to store hashes and metadata for integrity proof of medical-record changes. | No sustained implementation reported; because no document changes occurred, the core ledger-based change-monitoring function was not tested under real change events; limitations included short duration, small sample, and pediatric-clinic selection. |
| Cancer Gene Trust (CGT) (15) | UCSF Medical Center, United States; prospective oncology cohort | 18 consented patients; 17 with EHR data; cross-sectional data-sharing pilot | Data-completeness comparison and demonstration of sharing deidentified genomic, clinical, and imaging data. | Off-chain data storage with IPFS and Ethereum provenance hash of the data submission set. | Public app and data deposit reported, but no sustained operational use or downstream research uptake measured; limitations included reidentification risk, cross-institution patient linkage, missing OMOP fields, and manual registry coding. |
| decentralized biobanking, breast-cancer biobank (16) | Single NCI breast-cancer biobank, United States; live pilot | 1,080 enrolled; 405 app downloads/registrations; 140 NFTs minted and 125 claimed; approximately 10-week recruitment period | App-download and NFT-claim funnel, profile completion, specimen reconnection counts, minting cost, co-design, and cognitive-walkthrough feedback. | Blockchain/NFT element used to support donor recognition, transparency, and access to specimen-related information; specimen data and access functions also relied on conventional database and identity infrastructure. | Pilot relied on manual workflows and future API integration; barriers included gas fees, onboarding friction, manual linkage, scalability, and single-site demographic limitations. |
| MyHealthData / MyData platform (17) | 2 institutions, South Korea: Seoul National University Hospital and one private clinic | 1,228 sign-ups; survey n=976; October-December 2019 | Service-use counts and perceived usefulness, ease of use, and satisfaction. | Panacea public chain used to store hash values for integrity proof; record retrieval, FHIR conversion, visualization, and claims processing used API/relay-server infrastructure. | One-time survey without measured continuation; limitations included limited linked insurers, restricted data types, data capacity/transmission concerns, and interoperability across heterogeneous hospital information systems. |
| Clinical decision support/smart healthcare | | | | | |
| BRCA-CN consortium blockchain, BRCA variant interpretation (18) | Consortium of 6 clinical genetics laboratories, China; 24-month deployment | 66,485 variant submissions; 950 unique variants; 156 registered users; 89 active monthly users; 15-expert panel | Inter-laboratory concordance, VUS-rate reduction, ACMG compliance, report time, AI-vs-expert concordance, uptime, latency, and ClinVar concordance. | Permissioned consortium blockchain and smart contracts used to coordinate multi-institutional variant curation, preserve audit trails, record metadata/hashes, and support data-sovereignty requirements. | Closest to sustained operational use, with 24-month operation reported; limitations included six-lab scope, incomplete geographic representation, two-year observation window, and some advanced privacy features remaining aspirational. |
| Health data marketplace/monetization | | | | | |
| de-bi operational feasibility case study (19) | Same United States breast-cancer biobank/de-bi pilot as (16), reframed as an operational-feasibility case study | 1,080 enrolled over 10 weeks (Feb 16 to May 4, 2023); 930 (86%) confirmed biobank members; 405 app downloads/registrations; 148 with specimens; 140 NFTs minted, 125 claimed (89.3%); plus stakeholder workshops (n=10) and physician/scientist/biobanker engagement (n=9) | Stakeholder perceptions, biobank-enrollment change during pilot, biobank withdrawals, and app-activity funnel and biospecimen-tracking counts | Blockchain/NFT component used to support donor recognition, transparency, and a foundation for future decentralized biobanking and incentive models. | Real-world pilot with a measured institutional engagement effect. No sustained-use metric reported; communication, sustainability, governance, workflow balance, power dynamics, and institutional implementation remained open issues. |

**References**

1.	Cernian A, Tiganoaia B, Sacala I, Pavel A, Iftemi A. PatientDataChain: A Blockchain-Based Approach to Integrate Personal Health Records. Sensors. 2020 Nov 16;20(22):6538. doi:10.3390/s20226538

2.	Lo YS, Yang CY, Chien HF, Chang SS, Lu CY, Chen RJ. Blockchain-Enabled iWellChain Framework Integration With the National Medical Referral System: Development and Usability Study. J Med Internet Res. 2019 Dec 4;21(12):e13563. doi:10.2196/13563

3.	Khurshid A, Rajeswaren V, Andrews S. Using Blockchain Technology to Mitigate Challenges in Service Access for the Homeless and Data Exchange Between Providers: Qualitative Study. J Med Internet Res. 2020 Jun 4;22(6):e16887. doi:10.2196/16887

4.	Seaberg RW, Seaberg TR, Seaberg DC. Use of Blockchain Technology for Electronic Prescriptions. Blockchain Healthc Today. 2021 Oct 22. doi:10.30953/bhty.v4.183

5.	Bautista JR, Harrell DT, Hanson L, De Oliveira E, Abdul-Moheeth M, Meyer ET, et al. MediLinker: a blockchain-based decentralized health information management platform for patient-centric healthcare. Front Big Data. 2023 Jun 22;6:1146023. doi:10.3389/fdata.2023.1146023

6.	Bae YS, Park Y, Lee SM, Seo HH, Lee H, Ko T, et al. Development of Blockchain-Based Health Information Exchange Platform Using HL7 FHIR Standards: Usability Test. IEEE Access. 2022;10:79264–71. doi:10.1109/ACCESS.2022.3194159

7.	Despotou G, Evans J, Nash W, Eavis A, Robbins T, Arvanitis TN. Evaluation of patient perception towards dynamic health data sharing using blockchain based digital consent with the Dovetail digital consent application: A cross sectional exploratory study. Digit Health. 2020 Jan;6:2055207620924949. doi:10.1177/2055207620924949

8.	Yongjoh S, So-In C, Kompunt P, Muneesawang P, Morien RI. Development of an Internet-of-Healthcare System Using Blockchain. IEEE Access. 2021;9:113017–31. doi:10.1109/ACCESS.2021.3103443

9.	Motohashi T, Hirano T, Okumura K, Kashiyama M, Ichikawa D, Ueno T. Secure and Scalable mHealth Data Management Using Blockchain Combined With Client Hashchain: System Design and Validation. J Med Internet Res. 2019 May 16;21(5):e13385. doi:10.2196/13385

10.	Hirano T, Motohashi T, Okumura K, Takajo K, Kuroki T, Ichikawa D, et al. Data Validation and Verification Using Blockchain in a Clinical Trial for Breast Cancer: Regulatory Sandbox. J Med Internet Res. 2020 Jun 2;22(6):e18938. doi:10.2196/18938

11.	Huh KY, Jeong S un, Moon SJ, Kim MJ, Yang W, Jeong M, et al. METORY: Development of a Demand-Driven Blockchain-Based Dynamic Consent Platform Tailored for Clinical Trials. Front Med. 2022 May 13;9:837197. doi:10.3389/fmed.2022.837197

12.	Huh KY, Moon SJ, Jeong S, Kim M, Yang W, Jeong M, et al. Evaluation of a blockchain‐based dynamic consent platform (METORY) in a decentralized and multicenter clinical trial using virtual drugs. Clin Transl Sci. 2022 May;15(5):1257–68. doi:10.1111/cts.13246

13.	Mak BC, Addeman BT, Chen J, Papp KA, Gooderham MJ, Guenther LC, et al. Leveraging Blockchain Technology for Informed Consent Process and Patient Engagement in a Clinical Trial Pilot. Blockchain Healthc Today. 2021 Oct 14. doi:10.30953/bhty.v4.182

14.	Sung M, Park S, Jung S, Lee E, Lee J, Park YR. Developing a Mobile App for Monitoring Medical Record Changes Using Blockchain: Development and Usability Study. J Med Internet Res. 2020 Aug 14;22(8):e19657. doi:10.2196/19657

15.	Glicksberg BS, Burns S, Currie R, Griffin A, Wang ZJ, Haussler D, et al. Blockchain-Authenticated Sharing of Genomic and Clinical Outcomes Data of Patients With Cancer: A Prospective Cohort Study. J Med Internet Res. 2020 Mar 20;22(3):e16810. doi:10.2196/16810

16.	Sanchez W, Dewan A, Budd E, Eifler M, Miller RC, Kahn J, et al. Decentralized Biobanking Apps for Patient Tracking of Biospecimen Research: Real-World Usability and Feasibility Study. JMIR Bioinforma Biotechnol. 2025 Apr 10;6:e70463. doi:10.2196/70463

17.	Bae YS, Park Y, Kim T, Ko T, Kim MS, Lee E, et al. Development and Pilot-Test of Blockchain-Based MyHealthData Platform. Appl Sci. 2021 Sep 3;11(17):8209. doi:10.3390/app11178209

18.	Qu S, Liu R, Li S, Huang C, Zhang Y, Gao X, et al. BRCA-CN: a blockchain-based framework to support public variant databases sharing in multi-center community for diagnostic reference and China regulatory science. Hum Genet. 2025 Aug;144(8):877–98. doi:10.1007/s00439-025-02764-8

19.	Dewan A, Altiery De Jesus V, Budd E, Sanchez WL, Kahn J, Miller RC, et al. Decentralized Biobanking to Empower Patient Engagement in Biospecimen Research: Operational Feasibility Case Study. Biopreservation Biobanking. 2025 Oct 30;19475535251384429. doi:10.1177/19475535251384429
